# When the Safe Alternative Is Not That Safe: Tramadol Prescribing in Children

**DOI:** 10.3389/fphar.2018.00148

**Published:** 2018-03-05

**Authors:** Frédérique Rodieux, Laszlo Vutskits, Klara M. Posfay-Barbe, Walid Habre, Valérie Piguet, Jules A. Desmeules, Caroline F. Samer

**Affiliations:** ^1^Division of Clinical Pharmacology and Toxicology, Department of Anesthesiology, Pharmacology and Intensive Care, Geneva University Hospitals, University of Geneva Geneva, Switzerland; ^2^Department of Anesthesiology, Pharmacology and Intensive Care, Geneva University Hospitals, University of Geneva Geneva, Switzerland; ^3^Department of Basic Neuroscience, Faculty of Medicine, University of Geneva Geneva, Switzerland; ^4^Division of Anesthesiology, Unit for Pediatric Anesthesia, Children’s Hospitals of Geneva, Geneva University Hospitals, University of Geneva Geneva, Switzerland; ^5^Pediatric Infectious Diseases Unit, Department of Pediatrics, Children’s Hospital of Geneva, Geneva University Hospitals, University of Geneva Geneva, Switzerland; ^6^Anesthesiological Investigations Unit, Department of Anesthesiology, Pharmacology and Intensive Care, Geneva University Hospitals, University of Geneva Geneva, Switzerland; ^7^School of Pharmaceutical Sciences, University of Geneva, University of Lausanne Geneva, Switzerland

**Keywords:** tramadol, codeine, opioids, children, pharmacogenetics, CYP2D6, safety, pain

## Abstract

Children represent a vulnerable population in which management of nociceptive pain is complex. Drug responses in children differ from adults due to age-related differences. Moreover, therapeutic choices are limited by the lack of indication for a number of analgesic drugs due to the challenge of conducting clinical trials in children. Furthermore the assessment of efficacy as well as tolerance may be complicated by children’s inability to communicate properly. According to the World Health Organization, weak opioids such as tramadol and codeine, may be used in addition to paracetamol and ibuprofen for moderate nociceptive pain in both children and adults. However, codeine prescription has been restricted for the last 5 years in children because of the risk of fatal overdoses linked to the variable activity of cytochrome P450 (CYP) 2D6 which bioactivates codeine. Even though tramadol has been considered a safe alternative to codeine, it is well established that tramadol pharmacodynamic opioid effects, efficacy and safety, are also largely influenced by CYP2D6 activity. For this reason, the US Food and Drug Administration recently released a boxed warning regarding the use of tramadol in children. To provide safe and effective tramadol prescription in children, a personalized approach, with dose adaptation according to CYP2D6 activity, would certainly be the safest method. We therefore recommend this approach in children requiring chronic or recurrent nociceptive pain treatment with tramadol. In case of acute inpatients nociceptive pain management, prescribing tramadol at the minimal effective dose, in a child appropriate dosage form and after clear instructions are given to the parents, remains reasonable based on current data. In all other situations, morphine should be preferred for moderate to severe nociceptive pain conditions.

## Introduction

Effective pain management in children is essential but various factors make it difficult to achieve. The assessment of the analgesic efficacy as well as the toxicity are challenging because of the difficulty to communicate with small children. Furthermore, medication responses in children may differ from adults due to drug metabolism, ontogeny, and other-age related differences. Finally, therapeutic choices are limited by the lack of indication for a number of analgesic drugs due to the challenges to conduct clinical pharmacology studies in children and the little interest of the pharmaceutical industry.

The American Academy of Pediatrics (AAP) and other pediatric associations and academies have released guidelines on the management of nociceptive pain in children. The top 3 medications’ recommendations in children are paracetamol, ibuprofen, and opioids: non-opioids for mild nociceptive pain; non-opioids + weak opioids for moderate nociceptive pain and non-opioids + strong opioids for severe nociceptive pain. Codeine and tramadol are the only two opioids classified as weak opioids. In most countries, they do not require a restricted medical drug prescription and as “weak” opioids, they are often considered to have a lower potential for adverse drug reactions (ADR) than “strong” opioids.

Although mostly safe in adults and in children, neither paracetamol, ibuprofen, nor opioids are exempt from ADR and toxicity (Drendel et al., 2009). Since 2012, the European Medicines Agency (EMA) and the US Food and Drug Administration (FDA) have addressed safety concerns regarding codeine-containing drugs, due to life-threatening and fatal respiratory problems.

Codeine is a prodrug, that needs to be bioactivated. Its therapeutic effect relies on the fraction metabolized into morphine via the cytochrome P450 (CYP) 2D6, a polymorphic enzyme known for its great interindividual variability in activity. In CYP2D6 ultrarapid metabolizers (UM), the morphine-metabolized fraction can be much higher than average and lead to overdoses despite normal therapeutic doses intake (Kirchheiner et al., 2007; Crews et al., 2014). Both agencies now recommend avoiding codeine in children younger than 12 years regardless of the indication, and up to the age of 18 in patients undergoing tonsillectomy and/or adenoidectomy for obstructive sleep apnea, as well as in breastfeeding mothers (European Medicines Agency’s Pharmacovigilance Risk Assessment Committee [PRAC], 2015; US Food and Drug Administration, 2017).

Following this contraindication, tramadol replaced codeine in the analgesia management protocols as a substitute weak opioid in pediatrics. Tramadol is, however, another prodrug for its opioid activity metabolized by CYP2D6. Unlike the EMA, the FDA recently released a boxed warning regarding the use of tramadol in children who are obese, or have obstructive sleep apnea or severe lung disease, and made recommendations not to use tramadol in children younger than 12 years and in children younger than 18 years after ear-nose-and-throat (ENT) surgery (US Food and Drug Administration, 2017).

In order to better understand, in light of current concerns, whether tramadol is a safe alternative to codeine, this article aims to review tramadol pharmacokinetics (PK) and safety issues in children and to draw recommendations as well as suitable alternatives to decrease risks of ADR when treating moderate to severe nociceptive pain in children.

## History of Tramadol

Tramadol is a synthetic opioid designed in 1962 and marketed as Tramal^®^ in 1977 by Grunenthal GmbH. The total number of prescriptions per year in the United States in 2013 was estimated to be more than 44 million, making tramadol one of the most prescribed opioids (Patterson, 2017). Its labeled indication in adults is the treatment of moderate to severe nociceptive pain, whether acute or chronic. In children, tramadol’s labeled indication is not unanimous and varies from country to country. In Europe, tramadol is approved and licensed for use in children over 1–3 years of age, depending on the countries, for moderate to severe nociceptive pain management. In the United States, tramadol is only approved for children older than 17 years of age, but appears to be regularly used nevertheless (Friedrichsdorf et al., 2015; Stassinos et al., 2017). Despite a lack of precise data, it is recognized that its prescription has increased in children after codeine was contraindicated (Mahic et al., 2015).

## Pharmacokinetics

The PK of tramadol is well described in adults (Gong et al., 2014). However, as for many drugs, the PK of tramadol may not be simply extrapolated from the adult’s data due to ontogeny and other age-related differences (Kearns et al., 2003; Funk et al., 2012; Lu and Rosenbaum, 2014; Batchelor and Marriott, 2015; Allegaert et al., 2017). Although still limited, several studies have been carried out in children of different ages to assess its PK profile (Murthy et al., 2000; Payne et al., 2002; Allegaert et al., 2005, 2015; Garrido et al., 2006; Saudan and Habre, 2007; Vandenbossche et al., 2015).

**FIGURE 1:**
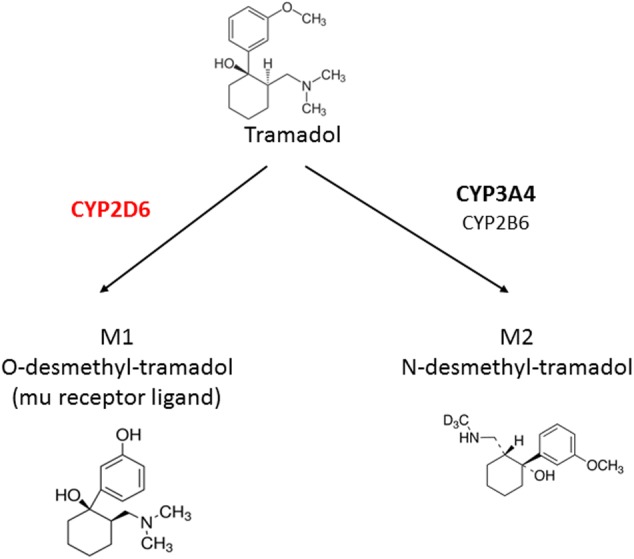
Metabolism of tramadol into its main metabolites.

After oral administration, tramadol is rapidly absorbed with a bioavailability of 75% in adults. Although estimated to be lower in neonates and small infants, tramadol’s bioavailability has not been clearly determined in children (Payne et al., 2002; Vandenbossche et al., 2015). Tramadol is rapidly distributed in the body, exhibits a limited protein binding of 20% and has an estimated volume of distribution of 2.7 L/kg in adults (Allegaert et al., 2015; Vazzana et al., 2015; Miotto et al., 2017). Due to age-modification in body composition, its volume of distribution is larger in children, particularly in preterm neonates where it is close to 4 L/kg (Allegaert et al., 2005, 2015; Saudan and Habre, 2007). Tramadol is extensively metabolized in the liver by *O*- and *N*-demethylations and by conjugation to form metabolites such as glucuronides and sulfates. The *O*-demethylation is mediated by CYP2D6 and converts tramadol to its main active metabolite, *O*-desmethyltramadol (M1). The *N*-demethylation to *N*-desmethyltramadol (M2) is catalyzed by CYP3A4 (major) and CYP2B6 (**Figure 1**; Gong et al., 2014). M1 is inactivated by glucuronidation, mostly via UGT2B7 and UGT1A8 (Lehtonen et al., 2010). A variety of *in vitro* and *in vivo* methods have been used to study the ontogeny of metabolic enzymes. Both CYPs and UGTs activities in neonates and small infants are known to be lower than in adults and to increase over time, each at its own speed, in an isoform-specific manner (Morselli et al., 1980; Alcorn and McNamara, 2002; Kearns et al., 2003; Blake et al., 2005; Allegaert et al., 2011). However, because of the difficulty in obtaining liver and other tissue material from children, data are rare and the rate at which the increase of individual enzyme activity occurs is still debated. It is generally agreed upon that CYP2D6 activity is already present at birth, rapidly increases over the first months and that final adults values are reached at 1 year of age, not later than 3 years of age (Treluyer et al., 1991; Tateishi et al., 1997; Allegaert et al., 2005, 2015). CYP3A4 activity is low to negligible at birth, then continuously increases in the first months of life to reach adult values at about 1 year of age (Tateishi et al., 1997; Johnson et al., 2006; Salem et al., 2014). In line with these observations, CYP2D6-mediated *O*-demethylation has been reported to develop sooner than CYP3A4-mediated *N*-demethylation (Allegaert et al., 2007).

The peak concentration (*C*_max_) of tramadol and its metabolite M1, occur at 1–2 and 3 h, respectively (Gong et al., 2014; Vandenbossche et al., 2015). The median time to the onset of action is 30 min to 1 h (Desmeules et al., 2003). After dose and weight normalization, the plasma concentration profile of tramadol and M1 appear lower in the pediatric population than adults (Gong et al., 2014; Vandenbossche et al., 2015).

M1 is transported from the blood into the liver cells via the organic cation transporter (OCT)-1 (Stamer et al., 2016). Little is known about ontogeny of drug transporters gene (Rodieux et al., 2016) but similarly to drug-metabolizing enzymes, they show specific rise in expression during organogenesis and after birth (Brouwer et al., 2015).

Tramadol and its metabolites are predominantly eliminated by the kidneys. Mean elimination half-life is about 5–6 h but greatly depends on CYP2D6 activity (Bastami et al., 2014). Elimination half-life of M1 is around 8 h. Renal elimination of both tramadol and M1 is slowed in infants until about 1 year of age which is compatible with kidney maturation (Bastami et al., 2014; Allegaert et al., 2015; T’Jollyn et al., 2015; Vandenbossche et al., 2015). From 1 year of age, total tramadol clearance and elimination half-life seem to be similar between children and adults (Murthy et al., 2000; Garrido et al., 2006; Allegaert et al., 2015).

## Pharmacodynamics

Tramadol exerts its analgesic activity via two complementary—opioids and non-opioids—mechanisms of action. The opioid activity results mainly from affinity binding of M1 metabolite to μ-opioid receptors. M1 affinity for the μ-opioid receptor is significantly higher than the parent drug affinity (approximately 700-fold; Gillen et al., 2000). The parent drug tramadol is furthermore a monoaminergic norepinephrine and serotonin transporter inhibitor, that increases the perisynaptic concentrations of serotonin and norepinephrine and enhances inhibitory effects on pain transmission in the spinal cord (Hennies et al., 1982; Kayser et al., 1992; Raffa et al., 1992, 1993).

The analgesic efficacy of tramadol has been established in several randomized, double-blinded, studies in adult patients with moderate to severe acute and chronic pain (Lee et al., 1993), and in a more limited number of studies in pediatric patients (Schaffer et al., 1986; Finkel et al., 2002; Rose et al., 2003; Akbay et al., 2010; Uysal et al., 2011; Alencar et al., 2012; Engelman and Marsala, 2012; Neri et al., 2013; Friedrichsdorf et al., 2015; Schnabel et al., 2015; Cooper et al., 2017; Wiffen et al., 2017). Most pediatric studies were conducted in the perioperative setting and in the management of acute pain. One study aimed to determine the efficacy of long-term use in children (Rose et al., 2003). After single or repeated (three to four times daily) doses of 1–3 mg/kg, tramadol was shown to be an effective analgesic in children (Finkel et al., 2002; Payne et al., 2002; Rose et al., 2003; Neri et al., 2013; Friedrichsdorf et al., 2015) and a 2015 Cochrane review (20 randomized controlled trials involving a total of 1170 patients) confirmed its efficacy in this population (Schnabel et al., 2015).

Only few safety and tolerability studies have been published on the long-term use of tramadol in children (Rose et al., 2003). The most common ADR, in both adults and children, after single and multiple doses are dizziness, nausea, confusion, drowsiness, tiredness, vomiting, constipation, headache, and dry mouth (Gibson, 1996; Rose et al., 2003; Moyao-Garcia et al., 2009). Respiratory depression, impaired consciousness, sedation, and seizure are rare, often related to tramadol overdose (Hassanian-Moghaddam et al., 2015; Tsutaoka et al., 2015; Stassinos et al., 2017). Due to its monoaminergic effects, tramadol increases the concentration of serotonin in the synaptic cleft and has been associated with serotonin syndrome in adults, in particular when combined with serotoninergic drugs (Beakley et al., 2015; Ansari and Kouti, 2016).

A large variability in interindividual responses to the same dose of tramadol has been observed, with respect to both therapeutic and ADR (Amanzio et al., 2001). Different factors may contribute to this variability, including body weight, sex, route of administration as well as drug–drug interactions or gene polymorphisms in metabolizing enzymes, transport proteins, and receptors related to the PK and pharmacodynamics (PD) of tramadol (Rollason et al., 2008; Samer et al., 2013; Gong et al., 2014; Lassen et al., 2015). Tramadol PD effects (analgesia and toxicity) partly depend on CYP2D6 activity, therefore drug–drug interactions and genes polymorphisms affecting CYP2D6 activity are of particular importance (Klotz, 2003; Zhou, 2009; Gong et al., 2014).

### Factors Modifying Clinical Responses to Tramadol

#### Drug–Drug Interactions

CYP2D6 accounts for only a small percentage of all hepatic CYPs (approximately 2–4%) but is the second most important CYP in term of drug metabolism (Rendic, 2002), metabolizing approximately 25% of currently marketed drugs (Ingelman-Sundberg et al., 2007). It is not considered inducible but can be strongly inhibited by a variety of drugs such as certain antidepressants (Benedetti, 2000). The level of consumption of CYP2D6 substrates or CYP2D6 inhibitors in children has not been specifically documented in the literature. Although probably much lower than in adults, it may not be negligible as the well-described CYP2D6 inhibitors risperidone and selective serotonin re-uptake inhibitors are increasingly prescribed in children and metoclopramide is often coadministrated with tramadol in the postoperative setting (Zito et al., 2003; Mitchell et al., 2008).

CYP2D6 inhibition has been shown to result in clinically significant failure to bioactivate tramadol along with a significant decrease of analgesic opioid efficacy (Tirkkonen and Laine, 2004; Laugesen et al., 2005), as it is the case with other prodrug opioids, such as codeine, hydrocodone, and oxycodone (Samer et al., 2010a; Monte et al., 2014). The consequence is, however, more complex with tramadol due to its dual mechanism of action. CYP2D6 inhibition does not only decrease the formation of M1; it also increases tramadol parent drug plasma concentrations which, in turn, may be associated with an increased risk of potentially life-threatening dose-dependent serotonin syndrome (Beakley et al., 2015).

CYP3A, which metabolizes tramadol to the M2 inactive metabolite, is sensitive to enzyme inhibition as well as induction. Carbamazepine and rifampicin, both potent inducers of CYP3A, have been shown to decrease tramadol analgesic effects shunting CYP2D6 and decreasing both tramadol and M1 concentrations (Klotz, 2003; Saarikoski et al., 2013). These data suggest that drug interactions involving CYP3A4 should also be taken into account and may modify the safety and efficacy profile of tramadol.

#### Pharmacogenomics

The highly polymorphic *CYP2D6* gene is one of the most investigated CYPs in relation to genetic polymorphisms. More than 100 allelic variants have been identified and this number is continuously growing. Many of these variants result in enzyme activity alteration (Ingelman-Sundberg et al., 2007). Four metabolizer phenotypes have been determined: (i) extensive metabolizers (EM) also referred to “normal” metabolizers who have normal enzymatic activity, (ii) UM who express more functional CYP2D6 enzyme than normal (30% carry gene duplications), (iii) intermediate metabolizers (IM) who express lower than normal amount of functional CYP2D6 enzymes, and (iv) poor metabolizers (PM) with little to no functional enzyme activity (carriers of tow deficient alleles or a gene deletion) (Zhou, 2009). EMs represent the high majority of the Caucasians as they account for 60–70% (Bradford, 2002). IMs account for 10–15% of Caucasians and up to 50% of Asians (Raimundo et al., 2000; Bernard et al., 2006). PMs represent 5–10% Caucasians, but are rare, <3%, or absent in other ethnic populations. UMs account for 2–6% of Caucasians but potentially up to 30% of Northern African and Arabian populations (Bradford, 2002; Llerena et al., 2014; Gaedigk et al., 2017). In the United States, ∼40% of the United States population is expected to carry one of the “extreme phenotype,” i.e., to be PM or UM (St Sauver et al., 2017).

The impact of *CYP2D6* polymorphism on opioids prodrugs disposition is well documented (Kirchheiner et al., 2007; Owusu Obeng et al., 2017). Patients with UM phenotype have increased plasma concentration of the active metabolite or an increased active metabolite/parent drug ratio, while PM phenotypes have decreased plasma concentration of the active metabolite or a decreased active metabolite/parent drug ratio (Pedersen et al., 2006; Gan et al., 2007; Stamer et al., 2007; Kirchheiner et al., 2008; Samer et al., 2010b; Andreassen et al., 2012; Stauble et al., 2014). Studies have shown a significant influence of *CYP2D6* polymorphism on tramadol opioid response and highlighted CYP2D6 UMs and PMs as “high risk phenotypes” for tramadol misuse, poor pain control and ADR (Stamer et al., 2007; St Sauver et al., 2017). It has been shown that CYP2D6 PMs have up to two times lower analgesic response rate (Pedersen et al., 2006; Stamer et al., 2007) and higher tramadol (up to 30% higher) and/or rescue medication consumption after surgery (up to two times higher) (Stamer et al., 2003, 2007; Slanar et al., 2012). CYP2D6 UMs with faster bioactivation of tramadol experience a higher frequency of nausea and a trend toward stronger miosis (Kirchheiner et al., 2008). Furthermore, different case reports have described near-fatal respiratory depression in UM patients treated with standard therapeutic doses of tramadol (Barnung et al., 1997; Stamer et al., 2008; Elkalioubie et al., 2011; Tantry et al., 2011). All had risk factors for respiratory depression or tramadol and its active metabolite accumulation, such as obesity or impaired kidney function.

Loss-of-function *SLC22A1* polymorphisms, encoding for OCT1, which concern ∼10% of the Caucasian population (Shu et al., 2003; Tzvetkov et al., 2013), have been associated with significantly higher plasma concentrations of M1 metabolite as well as longer lasting miosis and reduced tramadol need for pain control (Tzvetkov et al., 2011; Stamer et al., 2016).

Regarding PD genes, the μ-opioid receptor is encoded by the *OPRM1* gene. One of the most widely studied variant of *OPRM1* is the A118G base exchange (rs1799971) in exon 1, which results in reduced OPRM1 mRNA and protein levels (Zhang et al., 2005). G allele carriers present poorer response to opioid analgesia, including morphine and fentanyl (Romberg et al., 2005; Kasai and Ikeda, 2011). To date no study has clearly shown an impact of *OPRM1* polymorphism on the efficacy of tramadol.

## Clinical Use in Children

Tramadol is mainly used for acute nociceptive pain management in the context of trauma or in the postoperative setting (Neri et al., 2013; Friedrichsdorf et al., 2015; Schnabel et al., 2015; Yenigun et al., 2015; Ali et al., 2017; Liaqat and Dar, 2017; Maryam et al., 2017). It is also used, although less frequently, in the management of acute painful vaso-occlusive crisis in sickle cell disease (Erhan et al., 2007; Borgerding et al., 2013). Administration of tramadol for nociceptive chronic pain in children out of this context is rare.

## Tramadol, “Safety Concern” in Children

The safety of tramadol in children has been questioned since the mid-2010s, as respiratory depression induced by tramadol has been associated with overdosing but also with CYP2D6 UM phenotypes (Hassanian-Moghaddam et al., 2015).

On August 21, 2015, the FDA issued a first drug Safety Communication to alert health care providers and the lay public of the risk of respiratory depression in children receiving tramadol (US Food and Drug Administration, 2015). On April 20, 2017, the FDA issued a new drug safety communication with restrictions for the use of tramadol in children: i.e., a contraindication for treating pain in children younger than 12 years old and in children younger than 18 years old after surgery to remove the tonsils and/or adenoids, as well as a warning recommending against use of tramadol in obese adolescents between 12 and 18 years and children who have conditions such as obstructive sleep apnea or severe lung disease (**Table 1**; US Food and Drug Administration, 2017). From 2017, in the United States, codeine and tramadol use is thus now under the same restrictions.

**Table 1:** Latest FDA Drug Safety Announcement restricting the use of tramadol in children and breastfeeding women (US Food and Drug Administration, 2017).

• Contraindication: to treat pain in children younger than 12 years.
• Contraindication: to treat pain after surgery to remove the tonsils and/or adenoids in children younger than 18 years.
• Warning: to treat pain in adolescents between 12 and 18 years who are obese or have conditions which may increase the risk of serious breathing problems (obstructive sleep apnea or severe lung disease).
• Warning (strengthened): to treat pain in breastfeeding women due to the risk of serious adverse reactions in breastfed infants.

According to the FDA, their new warning was based on nine cases of respiratory depression, including three deaths reported in children under 18 years of age between 1969 and 2016 to the FDA Adverse Event Reporting System (FAERS; The Children’s Clinic, 2017; US Food and Drug Administration, 2017). Most of these episodes of respiratory depression had occurred within the first 24 h of tramadol administration. All three deaths involved children younger than 6 years of age. The doses were not known, but all of three cases of deaths reported supratherapeutic concentrations of tramadol (The Children’s Clinic, 2017; US Food and Drug Administration, 2017).

Aware of the limitations of spontaneous reporting systems for suspected ADR, we searched within the World Health Organization (WHO) pharmacovigilance database for recent tramadol ADR spontaneous reports, i.e., between 1992 and 2016. We found 15 individual case study reports of “*respiratory depression*” suspected to be attributed to tramadol in children. One report concerned a newborn, two reports concerned infants, three reports concerned children, and the majority, i.e., nine reports, concerned adolescents. All these reports were considered as “serious” and three led to the death of the patient. Routes of administration were often not reported, out of the 15 cases seven were oral, two intravenous, and one subcutaneous. The majority, i.e., 10 out of 15 involved accidental or intentional overdoses, and in many reports tramadol was not the only suspected drug. Only one report of suspected respiratory depression was attributed to oral tramadol alone, without documented overdose in a neonate (**Table 2**). Moreover, we found 14 individual case reports of “*death*” in children suspected to be linked to tramadol, adding up with the three deaths coded as “*respiratory depression*.” As for cases of respiratory depression, the great majority were described as accidental or intentional overdoses and other suspected drugs were involved (**Table 3**).

**Table 2:** Cases of respiratory depression in children suspected to be attributed to tramadol, spontaneously reported to the WHO pharmacovigilance database between 1992 and 2016.

Case	Age	MedDRA term	Documented overdose	Drug	Route of administration	Outcome
1	“0” Days	Respiratory depression Neonatal drug withdrawal syndrome		Tramadol	Oral	Recovered
2	8 Months	Respiratory depression Accidental exposure to product Grand mal convulsion Agitation, sedation, apneic attack Increased heart rate	Yes	Tramadol	Oral	Recovered
3	9 Months	Respiratory depression Somnolence	Yes	Tramadol	Oral	Recovered
4	3 Years	Respiratory depression Miosis, somnolence Attention disturbance	Yes	Tramadol	Oral	Recovered
5	3 Years	Respiratory depression		Tramadol Anesthetics Diclofenac Hydroxyzine	IV – PR –	Unknown
6	2 Years	Respiratory depression Overdose Accidental drug intake by child	Yes	Tramadol	Oral	Unknown
7	17 Years	Respiratory depression Overdose (intentional) Intoxication	Yes	Tramadol	–	Death
8	13 Years	Respiratory depression Bradycardia, somnolence		Tramadol Paracetamol Codeine	Oral Oral Oral	Recovered
9	17 Years	Respiratory depression Somnolence		Tramadol Oxazepam Alprazolam Methadone Diazepam Paracetamol Codeine	– – – – Oral – Oral	Recovered
10	17 Years	Respiratory depression Overdose Suicide Pulmonary edema	Yes	Tramadol	Oral	Death
11	15 Years	Respiratory depression Multiple drug overdose Bradycardia, hypotension, acidosis, somnolence, blood pH increase, abnormal behavior, blood potassium decreased, lethargy, toxicity to various agents, convulsion, reduced level of consciousness	Yes	Tramadol Chloramphenicol Methadone Cefdinir	– – – –	Unknown
12	17 Years	Respiratory depression Intentional drug misuse Multiple drug overdose (intentional) Reduced level of consciousness Blood pressure increased	Yes	Tramadol Cetirizine Risperidone Fluvoxamine	– – – –	Unknown
13	15 Years	Respiratory depression Pulmonary edema	Yes	Tramadol Pethidine Metamizole	SC SC IV	Death
14	13 Years	Respiratory depression		Tramadol	IV	Unknown
15	16 Years	Respiratory depression Somnolence, tachycardia	Yes	Tramadol Droperidol	IV IV	Recovered

*IV, intravenous; SC, subcutaneous; PR, per rectal; –, not known; MedDRA, Medical Dictionary for Regulatory Activities (a clinically validated international medical terminology dictionary (and thesaurus) used by regulatory authorities)*.

**Table 3:** Cases of death in children suspected to be attributed to tramadol, spontaneously reported to the WHO pharmacovigilance database between 1992 and 2016.

Case	Age	MedDRA term	Documented overdose	Drug	Route of administration	Outcome
1	11 Days	Death Non-accidental overdose Coma Respiratory arrest Drug level increased	Yes	Tramadol	Oral	Death
2	16 Months	Death Respiratory arrest		Tramadol Naproxen Oxycodone Diphenhydramine Cimetidine	Oral Oral Oral Oral Oral	Death
3	1 Month	Death Drug maladministration Drug level increased	Yes	Tramadol	Oral	Death
4	5 Years	Death Toxicity to various agents Cardiorespiratory arrest		Tramadol Morphine Diphenhydramine	Oral Oral Oral	Death
5	6 Years	Death		Tramadol Diphenhydramine	Oral Oral	Death
6	14 Years	Death Completed suicide	Yes	Tramadol Hydrocodone	Oral Oral	Death
7	14 Years	Death		Tramadol	–	Death
8	16 Years	Death		Tramadol		Death
9	15 Years	Death suicide	Yes	Tramadol Diclofenac Methylphenidate Paracetamol Salicylates	Oral Oral Oral Oral Oral	Death
10	15 Years	Death, fever, dyspnea, somnolence, dizziness, shock, anxiety, consciousness decreased, giddiness, hallucination		Tramadol Pseudoephedrine Loxoprofen Azelastine	IV Oral Oral Oral	Death
11	16 Years	Death suicide	Yes	Tramadol Ibuprofen Baclofen Lisinopril	Oral Oral Oral Oral	Death
12	17 Years	Death Completed suicide Cardiorespiratory arrest	Yes	Tramadol Risperidone Metaxalone Bupropion	Oral Oral Oral Oral	Death
13	16 Years	Death Drug overdose	Yes	Tramadol Infliximab	Oral IV	Death
14	12 Years	Death		Tramadol Paracetamol	Oral Oral	Death
1	17 Years	Respiratory depression Overdose (intentional) Intoxication	Yes	Tramadol	–	Death
2	17 Years	Respiratory depression Overdose Completed suicide Pulmonary edema	Yes	Tramadol	Oral	Death
3	15 Years	Respiratory depression Pulmonary edema	Yes	Tramadol Pethidine Metamizole	SC SC IV	Death

*IV, intravenous; SC, subcutaneous; –, not known; MedDRA, Medical Dictionary for Regulatory Activities (a clinically validated international medical terminology dictionary (and thesaurus) used by regulatory authorities)*.

In the literature, a single case report of a 5-year-old boy who developed respiratory depression after standard tramadol dose was published in 2015 (Orliaguet et al., 2015). He had a tonsillectomy for obstructive sleep apnea and was prescribed a standard pediatric dose of 1 mg/kg tramadol at home. Approximately 8 h after discharge, he was found lethargic, with pinpoint pupils, had episodes of apnea and an oxygen saturation of 48%. He fully recovered after naloxone. His M1 concentration (24 μg/mL) was higher than expected (Vandenbossche et al., 2015, 2016), and genotyping showed that he was an UM for CYP2D6 (Orliaguet et al., 2015).

## Perspective

Both tramadol and codeine are prodrug opioids bioactivated by CYP2D6 to exert their opioid analgesic effect, and changes in CYP2D6 activity (drug–drug interactions or genetic polymorphisms) have been shown to significantly alter the efficacy and safety of tramadol. Thus, despite limited data on serious tramadol’s casualties at appropriate therapeutic dosing in children, its safety is being legitimately questioned as a substitute for codeine. The risk-benefit ratio has to be weighed, in particular in children where the assessment of efficacy or ADRs is complicated by the inability of the patient to communicate properly.

Complete and detailed information to caregivers about the risks associated with tramadol administration and close monitoring of the child should allow adapting the treatment in case of inefficacy or ADR. This will, however, not always be sufficient to ensure safe and effective pain management and two alternative scenarios should be currently considered: (1) a personalized approach, which implies to identify patients at risk for “over or under response” and to adapt the dose according to CYP2D6 activity in order to preserve safe and effective use of tramadol; and (2) the choice of an alternative analgesic molecule.

### Personalized Approach

Two methods are currently available to assess the activity of CYP2D6: genotyping and phenotyping. CYP2D6 genotyping determines differences in the genotype of an individual by examining the individual’s DNA sequence from a blood or saliva sample, and can be done in children of all ages (once in a lifetime test). Phenotyping involves the oral intake of a probe drug metabolized by a specific CYP followed by a single measurement of plasma/capillary concentration ratio between the metabolite and the probe (metabolic ratio). This ratio defines an individual metabolic profile (Frank et al., 2007; Bosilkovska et al., 2014). Dextromethorphan is one of the gold standard probe drug for CYP2D6 and low dosing may be used to minimize the potential therapeutic effects of the probes and drug–drug interactions. In adults, a 10 mg dextromethorphan dose (i.e., at least two times lower than the standard therapeutic dose) is used (Bosilkovska et al., 2014, 2016). In children a dose of 0.15 mg/kg (i.e., two to four times lower than the standard therapeutic dose) has been successfully evaluated in our laboratory.

Genotyping and phenotyping are complementary methods and phenotyping offers the advantage of measuring the combined effects of genetic, environmental, endogenous, ontogenic, and other developmental factors. Combination of both methods is of particular interest in young children as genotype–phenotype relationships established in adults do not necessarily apply due to age-related developmental factors.

In the United States and around the world, government agencies are working on the implementation of pharmacogenetic testing in routine medical care and to develop standards for genetic testing laboratories (Hresko and Haga, 2012). Coverage by insurance companies is a critical step but has already been granted in some countries, such as the United States and Switzerland, when considered medically necessary to guide medical treatment or dosing. In the United States, reimbursement still depends on many factors such as the clinical context, the strength of evidence for a test, the specific medication as well as the insurance company (Hresko and Haga, 2012; Abul-Husn et al., 2014). In Switzerland, the pharmacogenetic tests, such as genotyping of CYP2D6, are covered by the health insurance when ordered by a medical specialist in clinical pharmacology.

Based on phenotypic and genotypic status, clinical dosing recommendations have been made available for prescribers. The Clinical Pharmacogenetics Implementation Consortium (CPIC^1^) and the Dutch Pharmacogenetics Working Group (DPWG) have indeed established codeine and tramadol dosing recommendations based on CYP2D6 activity (Swen et al., 2011; Dean, 2012; Crews et al., 2014). For tramadol, in CYP2D6 PMs, the recommendation is to select an alternative drug (not oxycodone or codeine) and/or to be extra alert to symptoms of insufficient pain relief; in CYP2D6 IMs, to be alert to decreased efficacy, to consider dose increase and if response is still inadequate to select an alternative drug (not oxycodone or codeine) and in CYP2D6 UMs, to decrease the dose by 30% and be alert for ADRs or to use an alternative drug (not oxycodone or codeine). These recommendations are validated in adults, and no specific guidelines have been specifically designed yet for the pediatric population. However, the St. Jude Research Hospital has already developed a clinical decision support system within the electronic health record to guide codeine prescribing in children starting at 9 months of age with sickle cell disease, based on the CPIC pharmacogenetics-based codeine prescribing adults recommendations and consistent with the FDA boxed warning (Gammal et al., 2016). Such decision tools could easily be translated to tramadol in order to enable safer administration of tramadol in the majority of patients.

### Choice of an Alternative Analgesic

The oral alternatives to tramadol and codeine are scarce. Paracetamol and ibuprofen cannot be considered as effective alternatives because of their insufficient efficacy to treat moderate to severe pain. Among the other opioids, oxycodone and hydrocodone are two opioid prodrugs bioactivated by CYP2D6 and the impact of CYP2D6 activity on their PD is now well established (Samer et al., 2010a,b; Stauble et al., 2014; Owusu Obeng et al., 2017). Buprenorphine is a partial μ-opioid receptor agonist, which differs from other opioids because of its “bell shaped” analgesic dose–response curve in animals (Lutfy and Cowan, 2004) and a potential ceiling effect limited by its partial agonist activity for PD effects (Walsh et al., 1994; Lutfy and Cowan, 2004). Currently, its administration cannot be considered in children, especially for acute nociceptive pain management, for various reasons. It has a poor gastrointestinal bioavailability (Brewster et al., 1981) and the available formulations of the drug (sublingual and transcutaneous) are not suitable in this setting. Sublingual route is indeed not appropriate in a young child and transcutaneous route does not allow adequate management of acute nociceptive pain which needs rapid titration. Furthermore PK and PD data are lacking and buprenorphine has not been approved in children. Tapentadol is, for some authors, the alternative of choice in children (Anderson et al., 2017). Its advantages are that its hepatic metabolism does not involve CYP2D6 but mainly glucuronidation and it has no active metabolites (Kneip et al., 2008). However, it is a recent drug, marketed in 2009 and is not yet indicated in children due to the lack of PK, efficacy and safety studies (Borys et al., 2015). Thereby morphine, despite being feared by most caregivers and parents, is for the moment the only available alternative. Morphine has demonstrated efficacy and safety when used appropriately in nociceptive pain management in children (Lynn et al., 1993; Wong et al., 2012). From the available literature, given at equianalgesic doses, morphine has not been associated with more side effects than other opioids (Kart et al., 1997). It can be used in children of all ages and is available in a variety of galenic forms making its administration easy in children.

## Conclusion/Recommendations

Tramadol, the current alternative to codeine in the pediatric population may not be as safe as initially thought for the same reasons as codeine.

In a perfect world, where pharmacogenetic profile would be instantly made available, we would recommend to adapt tramadol prescription to the patient’s pharmacogenetic profile. This is unfortunately not yet the case, and we thus recommend distinguishing the acute and the chronic nociceptive pain management settings.

In case of chronic and recurrent nociceptive pain or in situations where tramadol prescription can be planned in advance such as elective surgery, we strongly recommend that CYP2D6 phenotyping/genotyping are considered. Guidelines based on CYP2D6 genotype/phenotype for tramadol should be implemented, as with codeine.

In acute nociceptive pain and when the activity of CYP2D6 is unknown, it seems necessary to differentiate outpatients from inpatients who can be easily monitored 24 h before discharge. For inpatients it seems reasonable to continue tramadol prescription due to the presence of health care providers to monitor for ADR and efficacy. They should be clearly informed of the risks and able to monitor the child accordingly. In this setting, we advise starting at low doses (1 mg/kg/dose; three to four times daily) and adjusting the dosing according to tolerance and observed efficacy. At discharge, tramadol can be continued at the minimal effective dose depending on the tolerance and efficacy, if a child-friendly formulation is available, and after specific instructions are given to parents, orally but also in writing. The pediatric formulation would prevent dangerous overdoses and the parents’ instructions would allow parents to quickly detect the occurrence of ADR. In the outpatient setting, we would recommend to prefer morphine as a first-line therapy in all tramadol nave patients with unknown CYP2D6 status.

We further recommend to contraindicate tramadol and to prefer morphine in the following situations: immediate discharge after a surgical intervention, risk factors for respiratory depression (i.e., age < 1 year, sleep apnoea syndrome, obesity, ENT surgery), clinically significant drug–drug interaction, CYP2D6 extreme phenotypes, and/or documented inefficacy or intolerance to tramadol.

Tramadol, like all opioids, should be given with caution in children when initiating a treatment. However, in light of the current data, it appears that by supporting personalized medicine, good education of parents and caregivers as well as child-friendly formulation, its use may be pursued in the majority of the pediatric population for the treatment of moderate to severe pain.

Special and continued attention should be paid to pharmacovigilance data regarding tramadol administration in children and PK and PD studies evaluating other opioids in moderate or severe nociceptive pain in children, such as tapentadol.

## Author Contributions

FR and CS wrote the manuscript. JD, VP, LV, WH, and KP-B revised the manuscript.

## Conflict of Interest Statement

The authors declare that the research was conducted in the absence of any commercial or financial relationships that could be construed as a potential conflict of interest.

## Footnotes

1 https://cpicpgx.org/
